# The Supply of Calories, Proteins, and Fats in Low-Income Countries: A Four-Decade Retrospective Study

**DOI:** 10.3390/ijerph18147356

**Published:** 2021-07-09

**Authors:** Vasilii Erokhin, Li Diao, Tianming Gao, Jean-Vasile Andrei, Anna Ivolga, Yuhang Zong

**Affiliations:** 1 School of Economics and Management, Harbin Engineering University, Harbin 150001, China; basilic@list.ru (V.E.); gaotianming@hrbeu.edu.cn (T.G.); 2 School of Economics and Management, Wuhan University, Wuhan 430072, China; 2018301050015@whu.edu.cn; 3 Faculty of Economic Sciences, Petroleum-Gas University of Ploiesti, 100680 Ploiesti, Romania; andrei_jeanvasile@yahoo.com; 4 National Institute for Economic Research “Costin C. Kiritescu”, Romanian Academy, 050711 Bucharest, Romania; 5 Faculty of Social and Cultural Service and Tourism, Stavropol State Agrarian University, 355017 Stavropol, Russia; annya_iv@mail.ru

**Keywords:** calories, diet, fat, food intake, food security, income, proteins, trade

## Abstract

Over the past decades, both the quantity and quality of food supply for millions of people have improved substantially in the course of economic growth across the developing world. However, the number of undernourished people has resumed growth in the 2010s amid food supply disruptions, economic slowdowns, and protectionist restrictions to agricultural trade. Having been common to most nations, these challenges to the food security status of the population still vary depending on the level of economic development and national income of individual countries. In order to explore the long-run determinants of food supply transformations, this study employs five-stage multiple regression analysis to identify the strengths and directions of effects of agricultural production parameters, income level, price indices, food trade, and currency exchange on supply of calories, proteins, and fats across 11 groups of agricultural products in 1980–2018. To address the diversity of effects across developing nations, the study includes 99 countries of Asia, Europe, Latin America, the Middle East, and Africa categorized as low-income, lower-middle-income, and upper-middle-income economies. It is found that in low-income countries, food supply parameters are more strongly affected by production factors compared to economic and trade variables. The effect of economic factors on the food supply of higher-value food products, such as meat and dairy products, fruit, and vegetables, increases with the rise in the level of income, but it stays marginal for staples in all three groups of countries. The influence of trade factors on food supply is stronger compared to production and economic parameters in import-dependent economies irrelevant of the gross national income per capita. The approach presented in this paper contributes to the research on how food supply patterns and their determinants evolve in the course of economic transformations in low-income countries.

## 1. Introduction

Dietary patterns in various parts of the world are affected by a complex mix of variables that evolve over time. Since the 1980s, many countries of the world have demonstrated significant progress in improving the food security status of their populations and lifting people out of extreme poverty. Over a third of the world suffered from extreme poverty four decades ago, while only one-tenth of people lived below the international poverty line in the mid-2010s [1], the lowest level in recorded history [2]. Food security policies and interventions focused on agricultural production in the world’s poorest countries allowed to substantially increase food supplies, improve the quality of nutrition, and meet at least basic dietary energy needs [3]. The nutrient adequacy of diets became a core factor of food security and public health programs across the developing world.

The ways in which people modify their diets vary according to the income level [4,5,6], availability and affordability of healthy foods [7], domestic agricultural production and trade [8], stability of food supply [9], and many other determinants. Recent literature in the sphere of food security has emphasized the need for a complex approach to revealing how healthy diets are differently affected by a set of production, economic, and trade factors of food security [7]. Despite the efforts to combat malnutrition, the number of undernourished people has resumed growth in 2015 [10]. According to the estimations made by the Food and Agriculture Organization (FAO) and the World Health Organization [11], more than 1.3 billion people do not have regular access to nutritious and sufficient food, while over 690 million people suffer from hunger [3]. In 2020, the economic decline and disruption of food production and supply chains due to the COVID-19 pandemic have merely deepened the food insecurity problem, as the number of people suffering acute hunger appeared to double [12], while that of undernourished increased by at least 80 million people [3]. In many economies of Africa and Latin America, debt has increased significantly during the last decade [13], thus contributing to weakening growth prospects and challenging food security. If the negative trend continues, the FAO projects the number of undernourished people in the world to exceed 840 million in 2030 [3].

Lower incomes degrade dietary choices [8], as do agricultural production disruptions and food chain breakups [14]. In response to a degrading food security situation, people are forced to compromise on the nutritional quality of the food they consume, as well as reduce consumption. According to Laborde et al. [15], in low-income countries, poor households spend over 25% of their total income on staple foods and about half of their income on unprocessed nonstaple foods such as fruits, vegetables, and animal-source products. The diets become less diversified and less healthy as people cut consumption of pricier nonstaples. Recent systematic reviews and meta-analyses by Alarcon et al. [16] and Rousham et al. [17] report the increase in consumption of cheap, nutrient-poor, calorie-rich, and ultraprocessed foods not meeting international recommendations on salt, sugar, and fat levels [18]. These shifts in consumption patterns have large impacts on nutrition security and increase the risk of health consequences [19]. The achievements in the sphere of public health have remained rather modest in the least-developed and lower-income countries. At least 30% of the world’s population is affected by some form of malnutrition, including stunting, wasting, overweight, micronutrient deficiencies, or nutrition-related noncommunicable diseases (NCDs) [20]. Low-income economies of Africa and Asia concentrate about 94% of all stunted children in the world [3]. As found by Hirvonen et al. [21], dietary adjustments reduce the quality of nutrition and increase deficiencies of micronutrient consumption with potentially lasting adverse consequences for human health, especially for children, young people, and women of reproductive age [8]. For instance, the WHO [22,23] reports anemia becoming an increasingly challenging nutrition target to monitor globally.

Proceeding from the outlined tendencies in food consumption, this study aims to reveal how production, economic, and trade factors affect food supply patterns across a variety of country income groups and regions. The three hypotheses established in Section 2 test the specifics and major determinants of the supply of calories, proteins, and fats in diverse economic environments.

## 2. Theoretical Framework and Hypotheses

The theoretical framework employed in this study is based on the production–income–trade triangle that has been widely defined to be a cornerstone in the quality of diets in low-income countries [24,25]. Dual-process theories suggest that under economic stress, people tend to make no optimal food consumption choices [26]. Concerning healthy eating, this means that budgetary constraints could result in intuitive consumption decisions based on prices or physical availability of certain foods rather than their health benefits. Mackenbach [27] and Jetter et al. [28] acknowledge that the recognition of systematic determinants of food choices inspired by affordability and availability factors is crucial in understanding food supply patterns in poorer communities. Although some scholars have advocated for considering the systemic interactions of the three drivers of nutrient supply [7], this has been rarely done in relation to different country income groups within the same study. The FAO [3] reports that in lower-income countries, diets include more staple foods and less high-nutrient animal-source products, fruit, and vegetables compared to higher-income societies. According to Ranjit et al. [29] and Prabhakar et al. [30], food consumption habits and practices are less diversified among food-insecure low-income populations, where domestic sources of food supply prevail. Low-income countries rely more heavily on locally produced cereals, roots, tubers, and plantains (nearly 60% of aggregated food supply) than higher-income economies (only 22% of food supply). In view of such reliance, since the 1980s, nutrition improvement policies across the poorest countries have particularly emphasized the production of staple foods [31] with less attention given to the nutritional diet quality. As shown by Urgell-Lahuerta et al. [32], Malaiarasan et al. [33], and Herrera-Cuenca et al. [34], food intervention programs based on locally produced staples rarely diversify between people with health issues (diabetes, kidney disease, etc.) or population groups with special needs such as the elderly, pregnant women, and children. The per capita availability of cereals, roots, tubers, and plantains has increased driven by a rise in supply in Africa and some parts of Southeast Asia, but the quality of diets remains a problem. Therefore, it is safe to hypothesize that in Hypothesis 1.

**Hypothesis** **1.** *Low-income countries, diets are predominantly based on locally produced staples, while an adequate supply of calories and nutrients is mainly ensured by production factors.*

However, there remains the problem of poor year-round access to economically affordable sufficient diets in the majority of low-income communities. This puts a spotlight on nonproduction aspects of diet quality in the nutrition policy debate in developing countries. The income-related discrepancies in the per capita availability of foods have become particularly evident recently despite all efforts on combating economic inequality between and within countries. According to Beaulac et al. [35] and Hendrickson et al. [36], the evidence of poorer access to healthy food in lower-income and minority areas could be found even in developed countries. In the poorest communities, however, this problem has manifested itself most dramatically. Meira et al. [4], Rizky Maulidiana and Sutjiati [37], Smita et al. [38], and Carpio et al. [39] demonstrated the pivotal role of the level of income in establishing food consumption patterns in the least-developed economies. Wu et al. [40], Arce et al. [5], and Ranjit et al. [29] found economic factors to influence the intake of value-added nutrient-rich products, but such an effect could be expected. What is more striking is the dependence of staple intake on the level of income revealed by Sarica et al. [41], Sikandar et al. [42], and Qiu et al. [43].

Over the past decades, food security interventions have shifted from closing the dietary energy gap by means of increased production of staples to making diets more affordable in economic terms [44]. Malaiarasan et al. [33] and Urgell-Lahuerta et al. [32] demonstrated positive shifts in nutritional intake due to food interventions and price subsidies in low- and middle-income countries, irrespective of the actual income level of households. Due to a limited capacity to produce staple crops domestically, low-income economies are rather vulnerable to food inflation and other economic determinants of food consumption, such as per capita income [10]. According to Hussein et al. [45], nutrient intake in food-insecure least-developed countries is extremely sensitive to income shocks, especially for animal products which are the main sources of protein. However, the evidence of the economic factors–food supply relationship is inconclusive. Reeves et al. [46], Otsuka [47], Power [48], Esturk and Ören [49], Smith et al. [50], and Sonnino et al. [51], among others, demonstrated the likelihood of diets’ diversity and nutrient value to degrade with income inequality, while Elbushra and Ahmed [52], Ritchie et al. [53], and Thome et al. [54] found cointegration between economic parameters and food supply to be rather weak. According to the FAO [3], a key reason for growing food insecurity in developing countries is that many people cannot afford the increasing cost of healthy diets. The latter exceeds average food expenditures throughout Africa and Southeast Asia, where around 57% of the population cannot afford a healthy diet [3]. In lower-middle-income economies, where people are moderately food-insecure compared to low-income countries, typical diet composition is particularly determined by purchasing power of the population and prices of food and agricultural products. Nutritious foods are more expensive than energy-dense foods [55], and poverty constrains adequate access to healthy foods. Reducing food prices and increasing incomes can be an effective way of raising the overall supply of food and diversifying diets away from staples. Proceeding from this reasoning, we suggest the Hypothesis 2.

**Hypothesis** **2.** *The rise in a gross national income per capita stipulates a stronger influence of economic factors on food supply compared with that of production factors.*

In addition to low productivity, income inequality, and food inflation, food supply in many low-income countries critically depends on international trade. Integration of domestic supply chains into the global food market is widely viewed as a way to stabilize access to food in periods of domestic shortages [56,57]. It can also improve the availability of major foods by complementing the production capacity of the domestic agricultural sector [58,59]. Over the decades, food trade has become unprecedentedly liberalized within the World Trade Organization (WTO) multilateral framework and regional trade agreements. Since the 1980s, progressing globalization and trade have transformed food consumption patterns [60] and blended territorial diets. As shown by Drewnowski and Popkin [61] and Haddad et al. [62], dietary patterns in faster-growing developing economies have shifted more substantially from energy-dense staples to diets with a higher proportion of energy from animal-source foods. The standard conclusion drawn by many scholars, including Smith and Glauber [56], Yeung et al. [63], and Verter [64], among others, is that the importance of international trade in establishing sustainable food and nutrition security in developing countries is likely to increase in both the medium and long term. The FAO [3] observes most of the global increases in animal-source foods to occur in upper-middle-income countries, primarily, in Asia. The contribution from animal-source foods to a typical diet in upper-middle-income economies has reached 20% compared with only 11% in low-income countries [3]. Due to trade, territorial diets have become less linked to specific regions of the world [65], while in many low-income countries, food consumption habits have increasingly focused on added sugars and fats of high energy density and low nutritional value to the disadvantage of fruits, vegetables, and other forms of dietary fiber such as whole grains [66,67]. Since the early 2010s, the global agricultural market has been increasingly distorted by protectionist measures and trade restrictions imposed by major actors, such as the USA, Russia, and the EU [30,57]. Such policies contribute to distorting food supply chains, thereby threatening food security in low-income countries, many of which are net importers of food and agricultural products [68]. While lower-income countries rely on less-diversified, cheaper food imports [59], we assume as Hypothesis 3.

**Hypothesis** **3.** *Higher-income economies which are more deeply integrated into global food chains experience a stronger influence of trade factors on domestic food supply compared to production and economic determinants.*

Section 3 establishes the approach to testing the three hypotheses; Section 4 presents major results; Section 5 discusses findings through the supply–production, supply–income, and supply–trade lens; and Section 6 summarizes the test outcomes and outlines future research directions and potential policy implications of the study.

## 3. Materials and Methods

The methodology was built on revealing the effects of three categories of production, trade, and economic variables *X_n_* on the parameters of food intake *Y_n_* across low-income, lower-middle-income, and upper-middle-income economies in six regions of the world. The study employed the algorithm as specified in Table 1 and detailed in Section 3.1, Section 3.2, Section 3.3, Section 3.4 and Section 3.5.

### 3.1. Variables

The assessment of food supply quantity and diet quality has not been unified internationally. No composite index has been validated to measure the production, economic, and trade dimensions of diet quality [69,70]. One of the common approaches to measuring the food supply available for human consumption is to divide the respective quantity of primary agricultural products and the number of processed commodities by the population actually partaking in them. In terms of quantity, the FAO [71] reports data on food supplies as per capita daily calorie intake. FAO Food Balance Sheets (FBS) have been widely used as a proxy of the national average food supply to identify multiple aspects of dietary patterns in developing countries. For some low-income nations, the FBS is the only data source available for studying food consumption patterns over a long period of time. Therefore, we used the food supply dependent variable *Y*_1_ to estimate the average apparent intake of dietary energy in daily kilocalories per capita. A sufficient intake of calories to support energy balance for work and other activities every day is a benchmark of the energy-sufficient diet that is identified by the absolute lowest cost of meeting calorie needs from the cheapest starchy staple available in a country [3]. Alternatively, the nutrient-adequate diet provides not only a sufficient quantity of calories but also a qualitatively balanced mix of nutrients. To reflect the ability of a country’s food system to ensure affordability of major nutrients in the required proportions, we expressed the data on per capita food supplies in terms of protein (*Y*_2_) and fat (*Y*_3_) content (Table 2).

The overall calorie supply and protein and fat content were obtained from the FAO’s database [71] for 11 groups of food products out of 19 categories classified by the FAO/WHO Global Individual Food Consumption Data Tool [74] based on their nutritional relevance. The modifications were made to cater to the availability of data on *Y*_1–3_ variables in the entire period of 1980–2018, as well as the specifics of diets in particular regions included in the study. Thus, we included subcategories “Fruit, citrus” and “Fruit, tropical fresh” to *Y_n._*_2_ group and “Vegetables, leguminous” to *Y_n._*_3_ group and specified subcategories of meat in *Y_n._*_7_ group instead of “Red meat” and “Processed meat” used in the original FAO classification (Table 3).

Proceeding from the hypotheses established above, three categories of regressors were used to reflect the production (*X*_1–3_), economic (*X*_4–7_), and trade (*X*_8–10_) dimensions of effects on the intake of calories and nutrients. The food supply available in a country is the result of the total quantity of foodstuffs produced domestically (*X*_1_) added to the total quantity imported (*X*_9_) and adjusted to any change in supply that may have occurred because of export (*X*_8_) [3]. In developing economies, where a significant portion of GDP comes from primary sectors, the contribution of domestic production to food availability critically depends on the performance of the agricultural sector and its portion in the gross product (*X*_3_). In terms of the per capita food supply, the availability parameters are reflected by the gross per capita agricultural production index (*X*_2_). Low economic access to energy-sufficient and nutritionally adequate diets is one of the crucial factors of food insecurity. According to the FAO [3], costly and unaffordable healthy diets are associated with the increase in all forms of malnutrition not only in low-income countries but across the developing world, with over 1.5 billion people who cannot afford a diet that meets the required levels of essential nutrients. Therefore, we used income (*X*_4_) and price (*X*_5–7_) parameters to see how calories intake patterns have been transformed under the influence of economic factors.

### 3.2. Collinearity

When establishing multiple regression models, it is crucial to exclude regressors that strongly correlate with each other. This is done to eliminate the distorting effects of collinear variables on the regression analysis results. To ensure the robustness of the models, we employed the variance inflationary factor (VIF) test, one of the most commonly used approaches for measuring and reducing collinearity [75,76,77,78]. As recommended by Montgomery et al. [79], Kutner et al. [80], Snee [81], and Levine et al. [82], among others, when for a pair of regressors VIF > 5, such variables are considered collinear and excluded from the model. Following this guidance, at Stage 2, we checked sets of *X_n_* variables for collinearity and eliminated those for which the VIF exceeds 5. The calculation was performed for 10 multitudes of *X*_1–10_ regressors per country included in the study, a total of 990 calculations (see Section 3.4 for the list of countries).

### 3.3. Redundancy

At Stage 3, we proceeded with the resulting sets of non-collinear *X_n_* regressors and tested whether all of them yield low-redundant regression models. According to Johnsson [83], Zhou and Jiang [84], Evans [85], and Qi et al. [86], when regression analysis involves a significant quantity of data, regression models can be tested for redundancy without checking all sets of independent variables. As shown by Gupta [87], Hosmer et al. [88], and Sullivan and Wilson [89], this can be done by applying the stepwise regression technique. Among the stepwise regression variations, the best subsets approach (BSA) has gained particularly widespread acceptance in contemporary studies on multicollinearity [10,90,91] and prediction of interactions between variables in large arrays [92,93,94].

The BSA approach is based on the adjustment of *R*^2^ values in individual *Y–X* multitudes to account for the number of regressors and the sample size [82,95]. Due to the fact that at Stage 3 we compared non-collinear sets of *X_n_* variables with different numbers of regressors, the application of adjusted *R*^2^ instead of *R*^2^ was preferable. The goal was to identify the dataset with the largest adjusted *R*^2^, which was then used at Stage 4 for regression analysis. According to Ermakov et al. [96], Nikolov and Stoimenova [97], and Alshqaq and Abuzaid [98], such a goal can be achieved by using a criterion of Mallows’ *C_p_* statistic. Following the results of Hansen [99], Irurozki et al. [100], Liao and Zou [101], Feng et al. [102], and Aydin and Yilmaz [103], we utilized the *C_p_* criterion to measure the differences between the models constructed at Stage 2 and optimal (or true) models that best explain the correlations. The closer the *C_p_* is to the number of variables in a dataset, the more accurate the model would be (only random differences from the optimal model might occur) [10]. Thus, Stage 3 resulted in identifying the sets of variables where *C_p_* was close to or below (*k* + 1) (where *k* is the number of regressors).

### 3.4. Regression

Having checked the collinearity of *X_n_* variables and redundancy of the established multitudes, we proceeded with multiple regression analysis across all non-collinear regressors aggregated in 33 datasets separately for each of the three *Y_n_* (Table 2) in 11 food groups (Table 3). The *Y_n_* and *X_n_* parameters were aggregated from 1980 through 2018 from 99 countries categorized as low-income (22 countries), lower-middle-income (37 countries), and upper-middle-income economies (40 countries) according to the World Bank Country Classification [104]. Averaged values are presented in Appendix A, Table A1, Table A2 and Table A3, for *Y_n_* and Appendix B, Table A4, Table A5 and Table A6, for *X_n_*. To capture potential divergences in both calorie intake patterns from particular local foods and economic, production, and trade specificities in particular locations, we selected the countries from six regions of Africa, Asia, Latin America, and the Middle East (Table 4).

### 3.5. Strength and Direction of Relationships

The study was finalized by revealing the positive and negative impacts of *X_n_* regressors on *Y_n_* regressands separately for the three groups of countries and six regions. Based on the scale previously tested in Gao and Erokhin [10], positive effects were differentiated as highly positive (HP), positive (P), and marginally positive (MP); the negative ones were differentiated as extremely negative (EN), negative (N), and moderately negative (MN) (Table 5).

To determine to which of the six options *X_n_* falls, the two *X_max_* and *X_min_* extremes were excluded from the model (Equation (1)) and then the *X_mean_* value was calculated for each of the multitudes.

(1) $$X_{mean}=\frac{\sum X_{n}-X_{max}-X_{min}}{n-2}$$

The effects in *X–Y* multitudes were measured and classified for 990 sets of regressors and regressands (33 *Y_n_* (3 *Y* regressands and 11 food groups) and 10 *X_n_* regressors in 3 groups of economies).

## 4. Results

Collinearity was checked across *X*_1–10_ variables in 99 countries included in the study. Established regression multitudes were calculated with all ten regressors to find the variance inflationary factor values and eliminate those *X_n_* for which VIF exceeds 5 (or *R*^2^ > 0.8). Further application of the best-subsets stepwise regression to the remaining *X_n_* allowed us to check whether any of the collinear regressors were not detected by the VIF method (Table 6). Based on the adjusted coefficient of multiple determination and Mallows’ *C_p_* statistic, the best subsets of *X_n_* were selected out of competing multitudes for each country.

Having established non-collinear arrays of independent variables, we then performed multiple regression analysis for 99 countries (33 multitudes for each country) and generalized the results for the three categories of economies. 

In low-income economies, the production-related variables *X*_1–3_ exert prominent positive influence over all three regressands (Table 7). We register highly positive effects of gross agricultural production value (*X*_1_) and gross per capita agricultural production index (*X*_2_) on calorie and protein supply from cereals and tubers and fat supply from meat, dairy products, and animal and plant oils. This confirms our assumption that in low-income countries, food security is primarily ensured by the availability of domestically produced staples, such as cereals, roots, tubers, plantains, vegetables, and meat.

Compared to the production variables, the effects of economic and trade regressors on food, protein, and fat supply in low-income countries are both less pronounced and more heterogeneous. Calorie supply is moderately affected by the per capita value of the nominal gross national income. This could be explained by the overall low value of *X*_4_ and insignificant differences in this parameter across all countries included in the low-income group. Price-related parameters *X*_5–7_ are more influential, with negative extremes in major food groups (cereals, roots, tubers, and pulses). The strongest influence of economic parameters on the per capita supply of calories and nutrients is revealed in the SSA countries (Burkina Faso, Ethiopia, Guinea, Mali, Mozambique, and Sierra Leone), as well as in CA and MENA regions (Tajikistan, Yemen). This finding indicates that rising commodity price and consumer price indices substantially deteriorate calorie and nutrient supply across low-income countries as food inflation is tightly linked with poorer economic access to staples.

Trade positively affects food supply, particularly when we assess the import of value-added processed foods, including meat, milk, and dairy products. In most low-income countries, the domestic supply of a high-calorie diet is scarce, which is why imports appreciably improve food availability. In sub-Saharan Africa (Burkina Faso, Chad, Mali, Niger, and Tanzania), imports of food and agricultural products are identified to be effective at increasing the food supply. However, we see that currency exchange adversely affects economic access to imports, making food from abroad less affordable when local currency depreciates. Extremely negative effects of *X*_10_ on *Y*_n_ are most noticeable for higher-value meat and dairy imports. In the same vein, in lower-middle-income countries, economic effects are more prominent in higher-value food groups. In favor of Hypothesis 2, we see that the calorie supply in the “Meat and meat products” and “Milk and dairy products” categories is highly positively linked with the per capita value of nominal gross national income. Nevertheless, for lower-valued staples, the relationship between *X*_4–7_ and *Y_n_* is weaker (Table 8).

Similar to low-income countries, such a weak cointegration between economic variables and calorie supply in lower-middle-income economies could be explained by the high portion of locally produced staples in a diet. This effect is noticeable in the SA countries (Cambodia, India, Indonesia, Pakistan), where the portion of locally produced seasonal food in consumption is higher than in Africa and other regions included in the study. In Cote d’Ivoire, Nigeria, Zambia, Kenya, and some other SSA countries, the diversity of locally produced staples is narrower compared to Asia. When a portion of marketed food in supply is higher, economic parameters have a more meaningful effect in aggravating the quality of diets. Amid rising price indices and falling currency exchange rates, food purchases from abroad go down. The effects of *X*_5–7_ and *X*_10_ on the supply of calories, proteins, and fats are predominantly negative.

The strength of the *X*_8–10_–*Y_n_* relationship depends on the self-sufficiency degree of a country rather than the level of income. This confronts our suggestion that food supply in lower-middle-income economies is primarily affected by nontrade factors. In the countries where a large portion of the food availability in the domestic market is ensured by imports, currency exchange negatively influences the quantity of protein and fat supply. The stronger link between food supply parameters and trade-related variables in lower-middle-income economies compared to low-income countries stems from the fact that the former import higher-quality and pricier food products.

Upper-middle-income economies are more deeply integrated into global food supply chains compared to low-income and lower-middle-income countries. From this perspective, in furtherance of Hypothesis 3, the most significant causal relationships between trade-related variables *X*_8–10_ and the three regressands are found in this array of countries, especially in those with the highest value of nominal gross national income per capita (Brazil, Turkey, Russia, Colombia, Peru). As distinguished from the other two categories, in upper-middle-income economies, we see a prominent positive effect of food exports on the quantity of the supply of calories, proteins, and fats domestically (Table 9). This counterintuitive correlation can be explained by the role of food exports in establishing national income. Higher exports contribute to the growth in nominal gross national income per capita, which is then translated into stronger purchasing power in the domestic market and improved economic access of people to more qualitative and diverse diets [105].

Dismissing the suggestion that the influence of economic factors on calorie supply quantity diminishes with the growth in the level of income, the *X*_4_–*Y_n_* effect is found to be the strongest in upper-middle-income countries. It is highly positive not only for meat and dairy products, but also for lower-value staples, such as cereals, vegetable oils, and animal fats. This finding relates to the fact that in higher-income countries, diets are more diversified compared to lower-income economies, and the economic effects on calorie supply spread from high-value categories to a greater variety of foods, including staples. At the same time, while higher prices for staple foods can aggravate poverty traps in low-income countries, they have a less prominent effect on diets in relatively well-off households. The effects of *X*_5–7_ on *Y_n_* across upper-middle-income economies are more moderate compared to low-income and lower-middle-income countries.

## 5. Discussion

### 5.1. Hypothesis 1: Supply and Production

As acknowledged by the FAO and the WHO [11], “current food systems are being increasingly challenged to provide adequate, safe, diversified, and nutrient-rich food for all that contribute to healthy diets”. Changes in agriculture productivity [106,107], economic slowdowns and rises [57,108], and trade liberalization and protectionist restrictions on food exports and imports [109,110,111,112] help explain much of the observed transformations in the food supply in recent decades. Having been common to most nations, these challenges still vary depending on the level of economic development and income of individual countries. 

**Figure 1:**
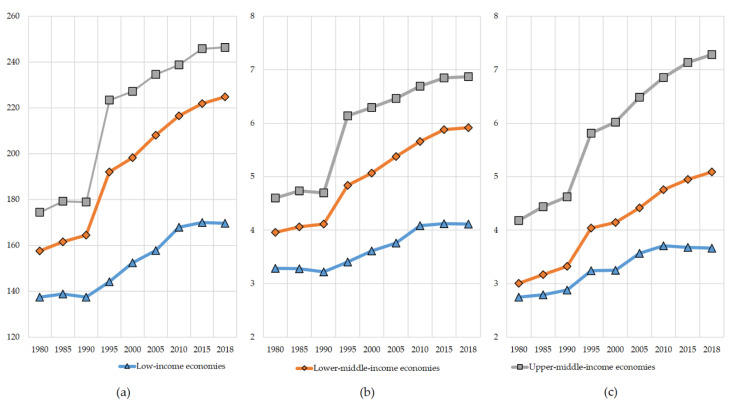
Changes in (**a**) food supply, kcal/capita/day; (**b**) protein supply, g/capita/day; and (**c**) fat supply, g/capita/day in low-income, lower-middle-income, and upper-middle-income economies in 1980–2018. Source: authors’ development.

Globally, food supply in terms of per capita value of calories, proteins, and fats has been on the rise since the 1980s, with the most significant improvement in the 1990s (Figure 1). It is hardly surprising that supply quantity is higher in wealthier countries compared to lower-income communities, but what matters is the dynamics of food supply parameters. Across low-income economies (in our array, mainly represented by the countries of sub-Saharan Africa), we see a distinct slowdown in protein supply quantity and even a decline in that of calories and fats in the last 8 to 10 years. The FAO [3] explains the decline in per capita food supply in low-income countries of Middle Africa and parts of Eastern Africa by a combination of civil conflicts in the continent (for instance, in the Central African Republic and Somalia) and a drop in crop yields because of climate variability. Both factors relate to degrading productivity of domestic agricultural sectors—due to either external disruptions or internal underperformance. In confirmation of Hypothesis 1, this well agrees with our finding of a highly positive and strong relationship in low-income countries between per capita food supply and production-related variables, such as gross agricultural production value, gross per capita agricultural production index, and agricultural sector’s share of GDP. The most significant correlations between *X*_1*–*3_ production variables and *Y_n_* are revealed in the Central African Republic, Chad, Gambia, Guinea-Bissau, and Sudan, the countries where the FAO [3] has been recording increases in undernourishment since the early 2000s. 

Cereal availability is highest in low-income countries among the three groups of economies included in the study (about 390 g/capita/day compared to 260 g/capita/day in upper-middle-income countries). This value has not changed much over decades, and the *X*_1–3_–*Y_n_* link has not weakened. For roots, tubers, and plantains and pulses, seeds, and nuts, we see a closer relationship between production factors and per capita calorie supply in the 2010s compared to the 1980–1990s. This goes in line with the FAO’s [3] projection of an increase in calorie supply in Africa by 2030 that would be achieved primarily by means of roots and pulses.

As demonstrated by Ma et al. [113] and Smith and Siciliano [114], an intensive increase in the production of staples in favor of present needs versus future yields has direct consequences on environmental degradation of land and other natural resources in developing countries. Thus, while contributing to the malnutrition fight by improving food availability in the market, local farmers compromise longer-term food security prospects. According to Galeana-Pizana et al. [115] and Van Wesenbeeck et al. [116], this particularly relates to smallholder agriculture through smaller farm size, lack of education among farmers, lower levels of trust in science, and time and labor constraints. Poorer yields of such staples as maize, sorghum, and groundnuts in the Great Lakes and the Horn of Africa areas [3] have particularly affected a stagnation of per capita calorie supply and rise in undernourishment in SSA countries in recent years. Diminished production performance is commonly addressed by applying more fertilizers. Increased use of chemical fertilizers by both farmers and large agricultural producers has played a vital role in improving food availability across the developing world, but fertilizer overuse has been deteriorating the environment and human health [116]. Alarcon et al. [16] term this threat “the double burden” in the sense that the existing malnutrition challenge is now being increasingly affected by food safety and environmental issues. The latter includes the formation of poisonous nitrates in groundwater [117], acidification of soils [118], higher emissions of acidifying substances and greenhouse gases [119], and plant uptake of hazardous micronutrients that are transmitted to human food and animal feed [120]. Therefore, improvements in the volume of food supply (Figure 1) do not necessarily result in healthier diets.

Government control means a lot to establishing more environmentally responsible food systems. However, the rigidity of regulations is very much compromised by the need to supply affordable food in sufficient quantity to people across all income groups. In upper-income countries, this “feed now–response tomorrow” dilemma could be addressed on the part of society by taking the “responsible eating” line. As Mihajlov and Puda [121] rightfully mention, modifying food choices towards healthier diets along with reducing food loss and waste can decrease greenhouse gas emissions and improve the production potential of food systems in the long run. While Piras et al. [122] found the largest share of food to be wasted at the household level in developed countries, the waste–consumption relationship is mediated by the level of income even across the developing world. For instance, Eini-Zinab et al. [123] revealed that eating more dairy products and fruits and vegetables and less bread, rice, pasta, legumes, hydrogenated fats, and sugars could reduce the total water and carbon footprints of the agricultural sector by 14% each. In lower-income communities, however, people struggle for eating rather than healthy eating, even less responsible eating. Rice, wheat, sugars, and other staples dominate food rations. We found highly positive effects of production variables on calorie supply from cereals (*Y*_1_), roots and plantains (*Y*_4_), and pulses (*Y*_5_). This finding confirms the estimation that low-income countries rely heavily on the domestic production of staple foods to combat undernourishment. As emphasized by Ecker and Hatzenbuehler [124], farmers in least-developed countries increasingly plant staples for their consumption at the expense of dietary diversification.

### 5.2. Hypothesis 2: Supply and Income

According to Vermeulen et al. [125] and Myers et al. [126], in addition to impacts on the quality and safety of food products, environmental shocks can affect prices and food availability. As we can see from the discussion above, food affordability reasoning is critical for making consumption decisions in lower-income communities. In higher-income ones, the perception of price in terms of the relationship between monetary value and health consequences is different. The “I am not rich enough to buy cheap products” behavior mode [121] does not work well for low-income countries, but it could benefit environment-responsible consumption in wealthier communities. While in low-income countries, cereals, roots, tubers, and plantains represent nearly 60% of all food available by weight, this percentage decreases with a rise in the level of economic development. In the studies from higher-income countries, Hanson and Connor [127], Johnson et al. [128], and Leung et al. [129], among others, have shown that both dietary diversity and consumption of highly nutritious products (food from animal sources, fruits, vegetables, etc.) tend to improve as the purchasing power of the population goes up. Green et al. [130] and Wu et al. [40] found food prices to have a greater effect on food consumption in low-income countries compared to other factors. Our findings, however, indicate stronger linkages between economic variables and per capita food supply in lower-middle-income countries than in low-middle economies. Thus, acknowledging previous results of Mundo-Rosas et al. [131], Ballard et al. [132], Vega-Macedo et al. [133], and other scholars, we can prove Hypothesis 2 by saying that both quantity and quality of calorie supply vary according to the income level of the country.

Nevertheless, we must admit that Hypothesis 2 is confirmed only for higher-value meat and dairy products, fruits, and vegetables, while for cereals, roots, and pulses, the relationship between *X*_4–7_ and *Y_n_* is weaker. This agrees with Stewart et al. [134] and Mackenbach [27], who recently linked the consumption of fruits and vegetables with the way households prioritize healthy eating. According to the FAO [3], the availability of fruits and vegetables in low-income and lower-middle-income has substantially increased since the 1990s, but it remains far below the 400 g/capita/day consumption target established by the WHO [135,136]. Only in upper-middle-income countries is this adequate food intake threshold met, but consumption of fruits and vegetables is distributed inadequately between regions. Thus, Hall et al. [137] and Afshin et al. [138] reported higher per capita values in Southeast Asia and lower supply quantity in Central Asia, Africa, and the Middle East. Upper-middle-income economies also demonstrate the most substantial increase in the amount of animal-source foods available.

The correlation between income (*X*_4_), cost of a diet (*X*_5–7_), and food security status of households has an important impact on the public health situation in a country [10,139]. Many scholars, including Herforth and Ahmed [140], Dizon et al. [141], Beydoun et al. [142], Grossman et al. [143], and Headey and Alderman [144], linked the cost and affordability of food and agricultural products with the quality and nutrition outcomes of a diet. Low intake of diverse complementary foods due to the economic unaffordability of an adequate supply can cause critical nutrient gaps in the diets [37,145,146]. Headey and Alderman [144] partially explained country-to-country differences in the prevalence of undernutrition and overweight among adults by varying levels of relative food prices.

Increased availability of animal-source products may have positive or negative implications for health depending on the context [3]. According to Hussein et al. [45], the extent to which protein-rich animal products such as meat and milk are consumed is extremely sensitive to income shocks. Exogenous influences, including food inflation, degrading purchasing power, and household budget constraints, are highly likely to result in a lower diversity of diets with less emphasis on meat and dairy consumption. Negative macronutrient implications have adverse consequences for levels of malnutrition. In low-income and lower-middle-income countries, where the impact of economic variables on *Y*_1–3_ is particularly strong, a tiny increase in the per capita supply of meat and other sources of quality protein can greatly improve the nutritional adequacy of diets. For higher-income communities, however, the effect of a continuing increase in consumption of meat above the healthy threshold could be ambiguous. For instance, Afshin et al. [138] associated increased consumption of red and processed meat in high-income countries with a risk of certain types of cancer [147] and other diet-related NCDs. Underconsumption of animal-source proteins and nutrients, on the contrary, can cause adverse health outcomes, including chronic conditions, mental health challenges, and increased risk of mortality, as found by Bakalis et al. [148], Garcia et al. [149], Berkowitz et al. [150], and Gundersen and Ziliak [151].

The finding that diet diversity improves with increasing level of income is consistent with the theoretical assumptions on consumer behavior previously formulated by Dorce et al. [152], Panait et al. [153], Chandra et al. [154], and Qiu et al. [43]. People who feel increasingly food secure due to a higher purchasing power are more certain about their ability to obtain higher-quality and more nutritious foods. Those who are increasingly food-insecure, on the contrary, are forced to compromise on the cost and quality of their diet and cut consumption of the most expensive (and healthier) products first [108,155,156]. Thus, Hirvonen et al. [21] and Van Hoyweghen et al. [157] revealed that reductions in household food consumption mainly reduced purchases of nutrient-dense foods. Shahzad et al. [158] proved convincingly that households handled the negative income shocks by eating less preferred food and getting support from government and charity organizations. The results of recent studies made by Swinnen and Vos [8] and Ceballos et al. [159] are also consistent with our finding of a stronger income–consumption link in wealthier communities compared to lower-income economies. Muhammad et al. [160] found that the purchase of pricier fruits and milk was particularly sensitive to changes in income and prices. According to Allee et al. [161], an increase in income directly influences an improvement in the food security status of the population, but the strength of this effect varies depending on the country. Thome et al. [54], Ritchie et al. [53], and Elbushra and Ahmed [52] explained the stronger influence of economic parameters on calorie supply in middle-income countries compared to low-income economies by a lower portion of locally produced staples in consumption. In our study, in confrontation with Hypotheses 2 and 3, the variability of the income–food supply relationship is found to be significant in both lower-middle-income and upper-middle-income countries with no decisive prevalence in the former.

### 5.3. Hypothesis 3: Supply and Trade

Recent food security literature commonly acknowledges the pivotal role of trade in ensuring access to nutrients for all at affordable prices through diversified international supply chains [9,56]. In the course of economic growth that has been progressing since the 1980–1990s across developing countries of Asia, Latin America, and some parts of Africa, many countries have been able to increase food purchases from abroad. This has substantially improved both the quantity of food supply and the diversity of diets in food-insecure communities [57,162]. Popkin [163], Bonaccio et al. [164], Popkin et al. [165], Annim and Frempong [166], and Lewis et al. [167], among others, show how diets shift away from staples towards more animal-source foods, sugars, fats, and oils with an increase in per capita income. Since the early 2000s, the largest increases in the availability of meat and dairy products, sugars, and fats have been observed in upper-middle-income countries. The FAO [3] acknowledges that the world’s most dramatic reductions in hunger in China and India have stemmed from long-term economic growth in both countries, agricultural development, and improved access to a variety of nutritious foods, including imports. Our study reveals the positive relationship between trade parameters *X*_8–10_ and the supply of calories, proteins, and fats in China to be one of the strongest among upper-middle-income countries. This finding correlates with the results of many studies that associate radical improvement in China’s food security with increasing integration of the country into international food supply chains [168,169,170]. A similar positive link between trade and improvements in the availability of food has been reported for many countries in Asia, Africa, and Latin America [9,57,59] in the sense of equalizing the quality of nutrition across the developing world and establishing a kind of what Hodges and Kimball [171] called “the global diet”. Most scholars, however, differentiate the effects of trade on food security from minor in least-developed countries to significant in upper-income economies [172,173]. Amid the backdrop of the overall positive correlation between trade variables and *Y_n_*, this income–trade–security pattern goes in line with our assumption that in higher-income economies, food supply more critically depends on trade factors rather than production or economic determinants.

There are, however, two exclusions that do not entirely favor Hypothesis 3. First is that a strong *X*_8–10_–*Y_n_* link is identified for some upper-middle-income economies, such as China, Russia, Turkey, Namibia, and Lebanon, but not all. For the remaining countries in this income group, the correlation is rather moderate (mainly marginally positive or moderately negative). On the other hand, distinct positive links between trade variables and food supply are revealed for many lower-middle-income countries, for example, Algeria, Tunisia, Cote d’Ivoire, Kyrgyzstan, and Uzbekistan. Overall, these findings are not consistent with the bulk of existing literature with respect to the strength of effects of international trade on food availability. Nevertheless, some studies, for instance, those of Hendrix et al. [174] and Wood et al. [68], revealed food import to be essential for meeting basic dietary needs in developing and least-developed countries. Frankenberg and Thomas [175] and Smith and Glauber [56] associated food price fluctuations in the global market with stronger adverse effects on the quality of diets in lower-income households compared to relatively well-off ones. Since the FAO [3] has been reporting continuous progress in improving per capita food supply in lower-middle-income countries such as Nepal, Pakistan, and Sri Lanka, the trade–supply pattern should be further checked. Our assumption was that the revealed duality in trade–supply relationships in lower-middle and upper-middle countries can be explained by varying degrees of dependence of domestic markets on agricultural imports. In relation to Hypothesis 3, this study resulted in finding stronger *X*_8–9_–*Y_n_* correlations in less self-sufficient countries that heavily depend on food supplies from abroad. Thus, it is safe to conclude that trade factors strongly affect the supply of calories, proteins, and fats in import-dependent economies regardless of the level of a gross national income per capita. Following Erokhin and Gao [10], Martin and Anderson [176], and Deuss [177], we assume that trade effects could be more pronounced in countries that particularly rely on supply from abroad to meet the demand for food in the domestic market. In such economies, disruptions in the food supply could have serious negative consequences for the nutritional security of the population. This agrees with Puma et al. [178], who found that LDCs suffer greater import losses due to disruption of food supply chains through their increased dependence on imports of staple foods. Currency depreciation drives up the cost of food imports; the effect of *X*_10_ on *Y_n_* is extremely negative in both lower-middle and upper-middle-income countries where food supply strongly depends on imports. Therefore, currency exchange becomes a factor of both food availability (more expensive imports due to currency depreciation) and access to food (the higher price of imported food on the domestic market when expressed in national currency) [10].

## 6. Conclusions

Diets in low-income countries evolve over time, being affected by diverse factors that interact in a complex manner to shape individual food availability and accessibility patterns in various income groups of economies in various regions of the world. In this study, we attempted to reveal the long-term trends in the influence of production, economic, and trade effects on the per capita supply of calories, proteins, and fats in low-income nations. The relationships between the regressands and corresponding regressors were discovered individually for 99 countries of East Asia and Pacific, South Asia, Europe and Central Asia, Latin America and the Caribbean, the Middle East and North Africa, and sub-Saharan Africa and generalized for the three income groups, given the alternations between the highest positive and most negative influences on the dependent variables. The consecutive application of the variance inflationary factor method, stepwise regression, best subsets approach, Mallows’ Cp statistic, and multiple regression method allowed us to identify the strengths and directions of effects of agricultural production parameters, income level, commodity and consumer price indices, food trade, and currency exchange on supply of calories and nutrients across 11 food groups.

### 6.1. Hypotheses Results

Three key findings emerged from the testing of the established hypotheses (Table 10).

- In low-income countries, food supply parameters are more strongly affected by production factors, such as agricultural production value, per capita agricultural production index, and the agricultural sector’s share of GDP.
- The effect of economic factors on the per capita supply of calories and nutrients increases with the rise in the level of income—it is the lowest in low-income countries and the highest in upper-middle-income economies. However, the effect is mainly tracked for higher-value food products, such as meat and dairy products, fruits, and vegetables, while it is marginal for staples.
- The effects of trade factors on food supply are stronger compared to production and economic determinants in import-dependent countries irrelevant of the income group they belong to. This effect is equally recorded in both lower-middle and upper-middle-income countries.

Food systems in low-income countries have been undergoing substantial changes in recent decades, but the evidence on how these transformations have affected nutritional security is limited. The results of testing these hypotheses demonstrate the necessity of taking an expanded view of food security, one that includes aspects of availability and affordability of adequate nutrient intake. Therefore, the contribution of the study to the literature could be expressed in the following three novel and essential outcomes:

First, such parameters as calorie, protein, and fat supply have not been comprehensively investigated against production, income, and trade variables. The effects on nutrient supply are assessed separately based on the established rating scale.

Second, the five-stage approach allowed for integration and clarification of the previously fragmented information on nutrient supply across diverse income patterns within the developing world. There have been studies on differences in food supply between developed and developing countries, but few scholars have captured the variation in economic factors between low-income, lower-middle-income, and upper-middle-income economies.

Third, the overlapping of the rating scale and income patterns provided the picture of strengths and directions of effects of agricultural production parameters, income level, price indices, food trade, and currency exchange on nutrient supply. In such a way, the study adds to the understanding of individual drivers of nutrition security across 99 countries.

### 6.2. Future Research Directions

Based on the obtained findings, we see potential implications of this study for further research on how food supply patterns and their determinants evolve in the course of economic transformations in low-income countries.

The set of production, economic, and trade variables used as regressors is open-ended. To capture a multidimensional character of food and nutrition security, it could be expanded by the parameters of stability of food supply and utilization of food and agricultural products. The five-stage regression analysis that involved collinearity checks based on the VIF and BSA methods resulted in establishing non-collinear regression models where regressands’ variations were well explained by regressors. However, due to the ongoing economic and social changes in low-income countries, a further focus on finding the most feasible influencing factors of the food supply could place the issue in the larger context of the quality and diversity of diets and their effects on public health in particular countries.

The projections on undernourishment may be substantially altered by differential impacts of production, economic, and trade factors on food supply across the regions. For the purpose of this study, we picked 22 low-income, 37 lower-middle-income, and 40 upper-middle-income countries, for which all *X_n_* and *Y_n_* data were available for the entire period of 1980–2018. Currently, the FAO provides information on calorie, protein, and fat supply quantity only at the national, aggregate level, not actual individual food or nutrient intake or the distribution of access to the food available by the different population groups. Therefore, in some cases, aggregated figures for income country groups might not fully reflect food supply specifics in individual countries or social groups. Both the array of countries and the time frame could be adjusted as more comprehensive data become available from the FAO, WHO, WTO, and other databases.

The environment-related effects of food production factors on nutrient supply addressed in the discussion should be further investigated. As demonstrated by many scholars [9,14,179,180], climate change, poor natural conditions, and fragile ecologies in many least-developed and developing countries make it difficult to combat malnutrition and simultaneously follow sustainable farming practices. For ensuring long-term food and nutritional security across the developing world, future studies must differentiate nutrient supply drivers between income patterns. The probing into the optimal use of fertilizers [116], liming [181], cultivation systems [115], food waste control [182,183], and environmentally responsible food consumption [121] should be conducted individually for lower- and upper-income countries in order to customize food policy measures.

### 6.3. Potential Policy Implications

Several policy recommendations could emerge from the study as well. The discussion of our results through the lens of healthy eating demonstrates that in low-income countries, this type of food consumption behavior is a matter of prioritization by policymakers rather than households. This is especially true for the poorest communities, where permanent nutrition insecurity presents serious health impacts. To make healthy diets more affordable, potential options should target every possible reduction in the final price of nutrient-rich products, including government support of shorter supply chains in meat and dairy sectors, increase in public investment in cleaner crop production, tax reliefs, and incentives for the application of organic fertilizers. In least-developed countries, price subsidies for fruits, vegetables, and meat can help the poor diversify their diets. As our findings showed diverse effects of economic and production factors on food supply, physical availability of food must be considered along with economic access. Subsidizing transaction costs of access points of sale could improve the availability of food in the most vulnerable communities. Furthermore, social safety nets and aid programs should be strengthened in the countries where the *X*_1–3_*–Y* relationship was found to be the strongest. In wealthier communities, where income-related effects on nutrient supply are less pronounced, we envision programs that could incentivize people to acknowledge the responsible eating concept. This includes a more conscious selection and use of food products, making consumption choices that involve locally grown food, and diversifying diets by introducing more fresh foods.

## Figures and Tables

**Table 1:** Study algorithm: stages, methods, and results.

Stage	Method	Results
Selection of variables		Establishment of the arrays of dependent (*Y_n_*) and independent (*X_n_*) variables
Collinearity check	Variance inflationary factor (VIF)	Elimination of *X_n_* variables with strong correlations between each other
Redundancy check	Stepwise regression, best subsets approach (BSA), Mallows’ statistic method (*C_p_*)	Determination of whether the resulting Stage 2 datasets all yield appropriate models with low redundancy
Regression analysis	Multiple regression method	Multiple regression analysis of all combinations of the selected non-collinear *X_n_* regressors aggregated in multitudes separately for each *Y_n_*
Strengths and directions of the *X–Y* relationships	Scale measurement of effects	Assessment of positive and negative impacts of *X_n_* regressors on *Y_n_* regressands separately for three groups of countries and six regions

Source: authors’ development.

**Table 2:** Regressands and regressors used in the study.

Index	Definition	Unit of Measure	Source of Data
**Regressands**			
*Y*_1_	Food supply, per food group	kcal/capita/day	FAO [71]
*Y*_2_	Protein supply quantity, per food group	g/capita/day	FAO [71]
*Y*_3_	Fat supply quantity, per food group	g/capita/day	FAO [71]
**Regressors**			
*X*_1_	Gross agricultural production value	USD 1 million (constant 2014–2016)	FAO [71]
*X*_2_	Gross per capita agricultural production index	Points (2014–2016 = 100)	FAO [71]
*X*_3_	Agricultural sector’s share of gross domestic product	%	UNCTAD [72]
*X*_4_	Nominal gross national income per capita	USD, current prices	UNCTAD [72]
*X*_5_	Commodity price index, food products	Points (2000 = 100)	UNCTAD [72]
*X*_6_	Commodity price index, agricultural raw materials	Points (2000 = 100)	UNCTAD [72]
*X*_7_	Consumer price index	Points (2010 = 100)	UNCTAD [72]
*X*_8_	Total exports of food and agricultural products	USD 1 million	UNCTAD [72], WTO [73]
*X*_9_	Total imports of food and agricultural products	USD 1 million	UNCTAD [72], WTO [73]
*X*_10_	Currency exchange rate	Local currency units/USD	FAO [71]

Source: authors’ development.

**Table 3:** Regressands and corresponding food group classifications used in the study.

Index	Food Groups	Food Subgroups
*Y_n._*_1_	Cereals and their products	Rice and rice-based products, maize and maize-based products, wheat and wheat-based products
*Y_n._*_2_	Fruits and their products	Fruit, fresh; fruit, citrus; fruit, tropical fresh
*Y_n._*_3_	Vegetables and their products	Vegetables, fresh; vegetables, leguminous
*Y_n._*_4_	Roots, tubers, plantains, and their products	Potato, sweet potato, and their products; cassava and its products; starchy roots (taro, yam) and their products; plantain and plantain-based products
*Y_n._*_5_	Pulses, seeds, nuts, and their products	Pulses (excluding soybeans) and their products, soybean and soy-based products (excluding soybean oil), nuts and their products, seeds and their products (excluding seed oil)
*Y_n._*_6_	Eggs and their products	Eggs, hen; eggs, other birds
*Y_n._*_7_	Meat and meat products	Meat, chicken; meat, pig; meat, cattle; meat offal, edible
*Y_n._*_8_	Fish, shellfish, and their products	Fresh and processed fish, cured fish, fresh and processed shellfish
*Y_n._*_9_	Milk and dairy products	Fresh milk, dried milk and subproducts, cheese, yogurt and other dairy subproducts
*Y_n._*_10_	Fats and oils	Vegetable fat and oil, animal fat and oil
*Y_n._*_11_	Sugars and sweeteners	Sugar and sweeteners, sugar crops

Source: authors’ development based on [3].

**Table 4:** Countries included in the study.

Regions	Low-Income Economies	Lower-Middle-Income Economies	Upper-Middle-Income Economies
East Asia and Pacific		Cambodia, Lao PDR, Mongolia, Myanmar, Philippines, Vietnam	China, Fiji, Indonesia, Malaysia, Thailand
Europe and Central Asia	Tajikistan	Kyrgyzstan, Moldova, Ukraine, Uzbekistan	Albania, Armenia, Azerbaijan, Belarus, Bosnia and Herzegovina, Bulgaria, Georgia, Kazakhstan, Russia, Serbia, Turkey, Turkmenistan
Latin America and the Caribbean	Haiti	Bolivia, El Salvador, Honduras, Nicaragua	Argentina, Belize, Brazil, Colombia, Costa Rica, Cuba, Dominica, Dominican Republic, Ecuador, Guatemala, Jamaica, Mexico, Paraguay, Peru, Suriname
The Middle East and North Africa	Yemen	Algeria, Egypt, Morocco, Tunisia	Iran, Iraq, Lebanon
South Asia	Afghanistan	Bangladesh, India, Nepal, Pakistan, Sri Lanka	Maldives
Sub-Saharan Africa	Burkina Faso, Central African Republic, Chad, Ethiopia, Gambia, Guinea, Guinea-Bissau, Liberia, Madagascar, Malawi, Mali, Mozambique, Niger, Rwanda, Sierra Leone, Sudan, Togo, Uganda	Angola, Benin, Cabo Verde, Cameroon, Cote d’Ivoire, Ghana, Kenya, Lesotho, Mauritania, Nigeria, Senegal, Tanzania, Zambia, Zimbabwe	Botswana, Gabon, Namibia, South Africa

Source: authors’ development based on [104].

**Table 5:** *X_n_* intervals and effects on *Y_n_*.

Intervals	Scale
(*X_max_* + *X_mean_*)/2 ≥ *X_mean_* ≥ (*X_min_* + *X_mean_*)/2	Positive (P)/Negative (N)
*X_max_* ≥ *X_n_* > (*X_max_* + *X_mean_*)/2	Highly positive (HP)/Extremely negative (EN)
(*X_min_* + *X_mean_*)/2 > *X_n_* ≥ *X_min_*	Marginally positive (MP)/Moderately negative (MN)

Source: adaptation from [10].

**Table 6:** Collinearity check and selection of the best subsets.

Country Group	Geographic Region	*R* ^2^	Adjusted *R*^2^	VIF	Mallows’ *C_p_* Statistic	Eliminated Regressors
Low-income economies	ECA	0.8096	0.7715	5.2521	8.1205	Tajikistan (*X*_2_, *X*_8_)
	LAC	0.7314	0.6940	3.7230	7.1473	Haiti (*X*_4_)
	MENA	0.7891	0.7806	4.7416	9.0018	Yemen (*X*_9_)
	SA	0.4027	0.3713	1.6742	4.7359	-
	SSA	0.6903	0.6057	3.2289	6.3967	Central African Republic (*X*_8_), Gambia (*X*_2_), Guinea (*X*_4_), Guinea-Bissau (*X*_8_), Liberia (*X*_1_), Malawi (*X*_8_), Mali (*X*_9_), Sudan (*X*_10_), Togo (*X*_8_), Uganda (*X*_2_)
Lower-middle-income economies	EAP	0.9146	0.7328	11.7096	3.4722	Cambodia (*X*_4_*, X*_8_), Lao PDR (*X*_10_), Mongolia (*X*_4_, *X*_8_, *X*_9_), Myanmar (*X*_2_, *X*_3_), Philippines (*X*_1*–*3_), Vietnam (*X*_5*–*7_)
	ECA	0.8592	0.8464	7.1023	6.1004	Kyrgyzstan (*X*_2_, *X*_4_), Moldova (*X*_9_), Ukraine (*X*_4_, *X*_5_), Uzbekistan (*X*_2_, *X*_4_, *X*_8_)
	LAC	0.7401	0.7157	3.8476	8.5263	Bolivia (*X*_7_), El Salvador (*X*_3_), Honduras (*X*_5_), Nicaragua (*X*_3_)
	MENA	0.7888	0.6895	4.7348	7.1950	Algeria (*X*_2_, *X*_9_), Egypt (*X*_8_), Morocco (*X*_9_), Tunisia (*X*_2_, *X*_8_, *X*_9_)
	SA	0.6749	0.6296	3.1104	5.2028	Bangladesh (*X*_8_), India (*X*_8_), Pakistan (*X*_2_, *X*_3_, *X*_8_), Sri Lanka (*X*_4_, *X*_5_)
	SSA	0.6927	0.5702	3.2541	9.0053	Angola (*X*_4_), Benin (*X*_5_), Cabo Verde (*X*_2_, *X*_10_), Cameroon (*X*_10_), Cote d’Ivoire (*X*_8*–*10_), Ghana (*X*_2_), Kenya (*X*_4_), Mauritania (*X*_3_), Nigeria (*X*_6_), Senegal (*X*_5_, *X*_6_), Tanzania (*X*_9_), Zambia (*X*_3_), Zimbabwe (*X*_4_)
Upper-middle-income economies	EAP	0.4063	0.3813	1.6844	9.6492	-
	ECA	0.7820	0.7054	4.5872	7.9667	Albania (*X*_2_, *X*_3_), Armenia (*X*_7_), Azerbaijan (*X*_3_), Belarus (*X*_4_), Bulgaria (*X*_6_, *X*_7_), Georgia (*X*_6_), Kazakhstan (*X*_3_), Russia (*X*_3_, *X*_4_), Serbia (*X*_4_), Turkey (*X*_8_), Turkmenistan (*X*_9_, *X*_10_)
	LAC	0.4234	0.3769	1.7343	10.4395	-
	MENA	0.5100	0.4870	2.0408	8.5941	-
	SA	0.7941	0.7837	4.8567	4.7206	Maldives (*X*_4_, *X*_5_)
	SSA	0.8738	0.8095	7.9239	6.6870	Botswana (*X*_4*–*6_), Gabon (*X*_3_), Namibia (*X*_8_, *X*_9_), South Africa (*X*_1*–*3_)

Note: *R*^2^ = coefficient of multiple determination; VIF = variance inflationary factor; EAP = East Asia and Pacific; ECA = Europe and Central Asia; LAC = Latin America and the Caribbean; MENA = Middle East and North Africa; SA = South Asia; SSA = sub-Saharan Africa. Source: authors’ development.

**Table 7:** The effects of *X_n_* on *Y_n_* in low-income economies.

Regressor	Food Groups										
	*Y_n._*_1_	*Y_n._*_2_	*Y_n._*_3_	*Y_n._*_4_	*Y_n._*_5_	*Y_n._*_6_	*Y_n._*_7_	*Y_n._*_8_	*Y_n._*_9_	*Y_n._*_10_	*Y_n._*_11_
Food supply											
*X*_1_	HP	P	P	HP	MP	MN	MN	P	MN	P	MP
*X*_2_	HP	MP	HP	HP	MN	N	MP	MP	N	P	P
*X*_3_	MP	MN	P	P	N	N	N	N	EN	MP	MP
*X*_4_	MN	MN	MN	MP	N	MP	MP	MN	MN	MN	MP
*X*_5_	EN	N	N	EN	MN	MN	MP	MN	MP	N	N
*X*_6_	N	MN	MN	EN	N	MP	P	MP	P	N	N
*X*_7_	EN	N	N	EN	EN	MN	MP	MN	MP	MN	MP
*X*_8_	MN	N	MP	MN	MP	MP	P	MP	P	MP	P
*X*_9_	P	MP	P	MP	MP	MN	HP	MP	HP	MN	MN
*X*_10_	P	P	MP	P	MN	MN	EN	N	EN	MN	MN
Protein supply quantity											
*X*_1_	P	MP	P	HP	P	MN	N	MP	MN	P	P
*X*_2_	HP	P	P	P	MN	MN	P	P	MN	MP	P
*X*_3_	MP	MN	MP	MP	N	N	MN	N	EN	P	MP
*X*_4_	N	N	N	MP	MN	MP	P	MN	N	MN	MP
*X*_5_	EN	EN	N	N	MN	N	MP	MN	MP	EN	EN
*X*_6_	N	MN	MP	EN	MN	P	MP	MP	P	MN	N
*X*_7_	EN	N	MN	EN	EN	MP	P	MP	MP	N	P
*X*_8_	N	MN	MP	MN	P	MP	MN	P	MP	MP	P
*X*_9_	MP	MP	MP	P	MN	MN	HP	MP	HP	MP	N
*X*_10_	MP	P	MN	MP	N	N	N	MN	EN	MP	N
Fat supply quantity											
*X*_1_	P	MP	MP	P	MN	MN	HP	P	HP	HP	MP
*X*_2_	MP	P	P	MP	N	MN	HP	MP	HP	HP	P
*X*_3_	MP	MN	MP	MN	MN	N	MP	P	P	MP	MN
*X*_4_	MN	N	N	MN	MN	MP	P	MN	MP	MP	MP
*X*_5_	N	MN	MN	N	MP	N	MP	MP	P	MP	MP
*X*_6_	MN	MP	MP	N	MN	P	MP	MP	P	MN	P
*X*_7_	N	MN	N	EN	N	MN	P	MN	P	MN	MP
*X*_8_	MN	MP	P	MN	MP	P	MP	MP	MP	P	MP
*X*_9_	MP	MP	MP	MP	P	MP	HP	P	HP	HP	P
*X*_10_	MN	MN	MP	MP	MN	N	EN	MN	EN	EN	N

Note: HP = highly positive; P = positive; MP = marginally positive; EN = extremely negative; N = negative; MN = moderately negative. Source: authors’ development.

**Table 8:** The effects of *X_n_* on *Y_n_* in lower-middle-income economies.

Regressor	Food Groups										
	*Y_n._*_1_	*Y_n._*_2_	*Y_n._*_3_	*Y_n._*_4_	*Y_n._*_5_	*Y_n._*_6_	*Y_n._*_7_	*Y_n._*_8_	*Y_n._*_9_	*Y_n._*_10_	*Y_n._*_11_
Food supply											
*X*_1_	P	HP	HP	MP	MN	MP	MN	HP	N	MN	MN
*X*_2_	MP	P	P	MP	N	P	MN	HP	MN	MN	N
*X*_3_	MN	MP	MP	MN	MN	N	N	P	MN	N	MN
*X*_4_	MP	P	MP	N	N	MP	HP	MP	HP	MP	P
*X*_5_	N	MN	MN	MP	MP	P	MP	N	EN	N	N
*X*_6_	EN	N	N	MP	P	HP	MP	N	EN	MP	MN
*X*_7_	N	MN	MN	MN	MP	P	MP	MN	EN	P	N
*X*_8_	MP	P	P	MN	N	MP	P	P	MN	MN	P
*X*_9_	HP	MP	MP	MP	MP	HP	HP	P	HP	P	MP
*X*_10_	N	MN	MN	MN	N	N	EN	MN	EN	N	MN
Protein supply quantity											
*X*_1_	HP	P	P	MP	N	MP	N	MP	MN	MN	MN
*X*_2_	MP	MP	MP	P	MN	P	MP	HP	N	N	N
*X*_3_	N	MN	MN	N	MN	MN	MN	P	MN	MN	MN
*X*_4_	MP	MP	P	MN	N	P	P	P	MP	HP	MP
*X*_5_	MN	MN	N	MP	P	MN	MP	MN	N	N	N
*X*_6_	EN	N	N	MP	MP	HP	HP	MN	N	MP	MN
*X*_7_	MN	N	MP	MP	P	HP	P	N	EN	MP	MN
*X*_8_	P	P	MP	N	MN	MP	MP	MP	MN	N	MP
*X*_9_	HP	P	MP	P	MP	P	P	MP	HP	HP	MP
*X*_10_	MN	MN	MP	MP	N	MN	EN	MN	EN	MN	N
Fat supply quantity											
*X*_1_	MP	P	P	MP	MN	MP	P	P	HP	HP	P
*X*_2_	MP	P	P	P	MN	MP	P	HP	HP	HP	P
*X*_3_	MN	MP	MP	N	N	MN	MN	MP	P	P	MP
*X*_4_	P	MP	MN	MN	MN	P	P	P	MP	MP	MP
*X*_5_	N	N	N	MP	P	MP	MP	MN	N	N	MN
*X*_6_	N	N	MN	P	MP	HP	HP	MN	MN	EN	N
*X*_7_	N	MN	MN	MN	P	MP	N	N	EN	EN	EN
*X*_8_	MN	MP	MP	MN	MN	MP	NP	MP	N	N	MN
*X*_9_	MN	MN	MN	MN	MP	HP	HP	P	HP	HP	P
*X*_10_	MN	MN	MN	MN	MN	N	EN	N	EN	EN	N

Note: HP = highly positive; P = positive; MP = marginally positive; EN = extremely negative; N = negative; MN = moderately negative. Source: authors’ development.

**Table 9:** The effects of *X_n_* on *Y_n_* in upper-middle-income economies.

Regressor	Food Groups										
	*Y_n._*_1_	*Y_n._*_2_	*Y_n._*_3_	*Y_n._*_4_	*Y_n._*_5_	*Y_n._*_6_	*Y_n._*_7_	*Y_n._*_8_	*Y_n._*_9_	*Y_n._*_10_	*Y_n._*_11_
Food supply											
*X*_1_	MP	MP	P	MN	MN	P	P	MP	P	MN	MN
*X*_2_	P	MP	MP	MN	MN	MP	P	P	P	MP	MP
*X*_3_	MN	MN	MN	N	N	MP	MN	MP	MP	P	MN
*X*_4_	HP	P	P	MP	MP	P	HP	HP	HP	MP	MP
*X*_5_	N	EN	EN	MN	MN	N	EN	N	EN	MN	N
*X*_6_	N	N	N	MN	MN	N	N	MN	N	MN	N
*X*_7_	N	N	N	MN	MN	N	EN	N	EN	MN	N
*X*_8_	P	HP	HP	MP	MP	MP	HP	HP	P	P	MP
*X*_9_	HP	P	P	P	P	P	HP	P	HP	MP	MP
*X*_10_	N	P	P	MN	MN	MN	P	MN	P	N	N
Protein supply quantity											
*X*_1_	P	MP	MP	MN	N	MP	HP	P	HP	MN	MN
*X*_2_	P	P	P	MN	N	MP	P	P	P	MN	MN
*X*_3_	MP	MP	MP	N	MN	MN	MP	MP	MP	N	N
*X*_4_	P	MP	MP	P	MP	MP	P	HP	HP	P	P
*X*_5_	MN	EN	N	MN	MN	N	N	MN	EN	MN	MN
*X*_6_	MN	N	MN	MN	N	N	MN	MN	MN	MP	MP
*X*_7_	N	MN	MN	N	N	MN	EN	MN	N	MP	MP
*X*_8_	P	HP	HP	P	MP	P	MP	P	MP	MP	MP
*X*_9_	P	MP	P	MP	MP	MP	HP	MP	P	MP	MP
*X*_10_	MN	P	MP	MN	MN	MN	MP	MN	P	MN	N
Fat supply quantity											
*X*_1_	MP	MP	MP	MN	MN	P	HP	MP	HP	HP	P
*X*_2_	P	P	MP	N	MN	P	MP	P	P	P	MP
*X*_3_	MP	MP	MP	N	MN	MP	MP	MP	MP	MP	MP
*X*_4_	P	MP	MN	MN	MP	P	HP	P	HP	HP	P
*X*_5_	MN	MN	MN	MN	MP	N	EN	N	EN	EN	N
*X*_6_	MN	MP	MP	MN	MN	N	EN	N	N	N	MN
*X*_7_	MP	P	MN	MN	N	MN	N	N	EN	N	MN
*X*_8_	P	P	P	P	MP	MP	P	MP	P	P	MP
*X*_9_	MP	MP	MP	MP	P	P	HP	HP	HP	HP	P
*X*_10_	MN	MN	MN	MN	MN	N	N	N	MN	N	MN

Note: HP = highly positive; P = positive; MP = marginally positive; EN = extremely negative; N = negative; MN = moderately negative. Source: authors’ development.

**Table 10:** Summary of production, economic, and trade effects on the supply of calories, proteins, and fats in the three income groups of countries in 1980–2018.

Factor/Parameter	Effects/Income Groups				
	Low-Income	Lower-Middle-Income	Upper-Middle-Income	Hypothesis	
Production factors					
Calorie supply	Strong	Weak	Weak	Confirmed	
Protein supply	Moderate	Moderate	Weak		
Fat supply	Strong	Weak	Moderate		
Economic factors					
Calorie supply	Moderate	Strong	Moderate	Partly confirmed for higher-value food products	
Protein supply	Strong	Strong	Moderate		
Fat supply	Weak	Moderate	Strong		
Trade factors					
Calorie supply	Weak	Moderate	Strong	Partly confirmed for import-dependent countries	
Protein supply	Weak	Weak	Strong		
Fat supply	Moderate	Strong	Weak		

Source: authors’ development.

## Data Availability

The data presented in this study are available on request from the corresponding author.

## Appendix A

**Table A1:** Food supply (*Y*_1_) per country categories and food groups in 1980–2018, kcal/capita/day.

Index	Food Group	1980	1985	1990	1995	2000	2005	2010	2015	2018
Low-income economies										
*Y*_1.1_	Cereals	630.773	685.000	670.409	738.591	774.136	780.273	861.500	882.864	884.136
*Y*_1.2_	Fruits	104.727	102.182	97.091	98.045	90.545	89.909	87.182	85.727	81.455
*Y*_1.3_	Vegetables	23.136	22.091	22.909	26.091	25.364	28.045	27.727	35.545	37.773
*Y*_1.4_	Roots, tubers, plantains	302.000	270.818	266.409	231.273	280.318	276.682	275.545	272.045	265.545
*Y*_1.5_	Pulses, seeds, nuts	91.182	79.818	78.000	75.955	84.364	93.273	108.909	109.773	107.364
*Y*_1.6_	Eggs	2.727	2.955	3.045	3.045	2.864	3.455	3.682	4.091	3.955
*Y*_1.7_	Meat	61.273	59.227	60.727	63.773	67.682	71.000	74.364	73.182	72.818
*Y*_1.8_	Fish, shellfish	12.455	10.727	11.500	10.864	11.091	12.455	13.955	14.591	14.909
*Y*_1.9_	Milk and dairy products	48.864	48.045	47.318	53.182	54.455	63.045	67.136	59.364	60.500
*Y*_1.10_	Fats and oils	154.955	160.864	168.000	192.000	187.000	206.227	212.045	212.091	212.045
*Y*_1.11_	Sugars and sweeteners	80.227	84.727	86.682	92.591	99.500	111.773	114.682	120.909	125.273
Lower-Middle-income economies										
*Y*_1.1_	Cereals	983.703	1012.757	1022.432	1172.865	1181.351	1223.243	1238.135	1262.378	1269.000
*Y*_1.2_	Fruits	68.595	64.703	58.568	70.135	69.486	78.270	85.838	96.595	99.459
*Y*_1.3_	Vegetables	23.081	24.270	25.676	32.351	40.703	45.973	54.243	62.486	62.405
*Y*_1.4_	Roots, tubers, plantains	148.162	147.703	155.216	180.459	188.811	198.541	208.189	210.270	213.486
*Y*_1.5_	Pulses, seeds, nuts	51.405	54.162	56.027	55.297	61.270	68.054	74.838	70.486	72.351
*Y*_1.6_	Eggs	6.973	7.189	7.378	9.595	10.108	11.568	13.324	15.054	15.216
*Y*_1.7_	Meat	71.622	72.649	77.459	97.703	103.595	112.486	127.676	137.324	138.838
*Y*_1.8_	Fish, shellfish	15.162	16.811	16.730	16.378	20.027	22.297	25.297	27.730	27.243
*Y*_1.9_	Milk and dairy products	61.297	62.649	60.514	91.270	99.027	106.649	111.622	118.676	119.135
*Y*_1.10_	Fats and oils	151.541	164.081	175.351	208.946	208.676	221.919	237.757	241.054	252.405
*Y*_1.11_	Sugars and sweeteners	151.324	150.405	153.378	178.378	197.568	199.378	204.730	199.081	202.757
Upper-middle-income economies										
*Y*_1.1_	Cereals	863.450	885.575	876.200	1115.475	1125.350	1113.275	1115.250	1139.375	1134.750
*Y*_1.2_	Fruits	97.300	97.725	94.500	109.650	114.350	118.200	121.725	133.450	135.750
*Y*_1.3_	Vegetables	29.700	32.925	35.050	51.075	57.325	66.800	69.700	71.850	71.350
*Y*_1.4_	Roots, tubers, plantains	110.900	109.750	104.525	134.725	133.600	143.050	139.800	147.700	142.975
*Y*_1.5_	Pulses, seeds, nuts	56.500	55.375	53.975	55.075	51.025	54.975	51.625	51.075	51.225
*Y*_1.6_	Eggs	16.300	16.975	18.725	24.225	25.300	28.950	31.800	34.850	36.375
*Y*_1.7_	Meat	129.450	137.000	140.975	190.950	197.125	206.550	230.800	234.525	240.550
*Y*_1.8_	Fish, shellfish	23.300	26.150	22.400	27.575	31.725	32.550	35.375	34.075	31.275
*Y*_1.9_	Milk and dairy products	106.600	102.875	107.350	160.725	166.400	173.925	176.875	184.200	185.950
*Y*_1.10_	Fats and oils	210.325	229.650	242.375	280.750	290.050	319.900	333.475	348.525	357.500
*Y*_1.11_	Sugars and sweeteners	274.825	278.200	272.325	307.675	306.550	323.000	320.175	324.000	323.300

Source: authors’ development.

**Table A2:** Protein supply quantity (*Y*_2_) per country categories and food groups in 1980–2018, g/capita/day.

Index	Food Group	1980	1985	1990	1995	2000	2005	2010	2015	2018
Low-income economies										
*Y*_2.1_	Cereals	15.098	16.404	16.115	18.122	18.995	19.095	20.911	21.435	21.465
*Y*_2.2_	Fruits	1.187	1.152	1.096	1.103	1.029	1.020	1.005	0.960	0.909
*Y*_2.3_	Vegetables	1.263	1.210	1.224	1.340	1.279	1.380	1.352	1.715	1.791
*Y*_2.4_	Roots, tubers, plantains	2.928	2.705	2.628	2.474	3.033	3.026	3.044	3.049	2.989
*Y*_2.5_	Pulses, seeds, nuts	5.953	5.184	5.049	4.928	5.464	6.044	7.043	7.090	6.917
*Y*_2.6_	Eggs	0.227	0.236	0.256	0.254	0.238	0.278	0.304	0.335	0.319
*Y*_2.7_	Meat	4.765	4.598	4.586	4.610	4.953	5.271	5.507	5.249	5.321
*Y*_2.8_	Fish, shellfish	1.991	1.705	1.846	1.749	1.770	1.971	2.211	2.346	2.366
*Y*_2.9_	Milk and dairy products	2.717	2.805	2.591	2.848	2.901	3.262	3.480	3.109	3.157
*Y*_2.10_	Fats and oils	0.027	0.027	0.029	0.029	0.028	0.030	0.035	0.030	0.032
*Y*_2.11_	Sugars and sweeteners	0.010	0.007	0.005	0.005	0.003	0.003	0.003	0.003	0.003
Lower-middle-income economies										
*Y*_2.1_	Cereals	24.586	25.218	25.418	29.670	29.751	30.547	30.780	31.469	31.608
*Y*_2.2_	Fruits	0.768	0.736	0.667	0.771	0.761	0.881	0.968	1.091	1.114
*Y*_2.3_	Vegetables	1.159	1.211	1.270	1.550	1.946	2.153	2.531	2.895	2.879
*Y*_2.4_	Roots, tubers, plantains	1.818	1.823	1.894	2.327	2.540	2.787	2.960	2.948	3.004
*Y*_2.5_	Pulses, seeds, nuts	3.332	3.502	3.632	3.578	3.969	4.409	4.832	4.535	4.667
*Y*_2.6_	Eggs	0.558	0.569	0.586	0.767	0.811	0.917	1.053	1.188	1.201
*Y*_2.7_	Meat	5.309	5.299	5.562	6.816	7.349	8.023	9.012	9.680	9.742
*Y*_2.8_	Fish, shellfish	2.395	2.641	2.615	2.560	3.108	3.437	3.916	4.276	4.211
*Y*_2.9_	Milk and dairy products	3.520	3.633	3.515	5.115	5.432	5.912	6.091	6.480	6.542
*Y*_2.10_	Fats and oils	0.043	0.042	0.048	0.056	0.061	0.073	0.079	0.089	0.090
*Y*_2.11_	Sugars and sweeteners	0.034	0.031	0.024	0.021	0.020	0.020	0.021	0.018	0.016
Upper-middle-income economies										
*Y*_2.1_	Cereals	22.094	22.713	22.435	29.201	29.295	28.766	28.787	29.362	29.232
*Y*_2.2_	Fruits	1.117	1.113	1.098	1.258	1.312	1.333	1.375	1.525	1.561
*Y*_2.3_	Vegetables	1.376	1.516	1.566	2.293	2.538	2.934	3.099	3.185	3.166
*Y*_2.4_	Roots, tubers, plantains	1.647	1.650	1.568	2.323	2.287	2.490	2.396	2.485	2.436
*Y*_2.5_	Pulses, seeds, nuts	3.612	3.538	3.439	3.520	3.274	3.526	3.309	3.277	3.287
*Y*_2.6_	Eggs	1.263	1.315	1.447	1.870	1.956	2.239	2.454	2.682	2.799
*Y*_2.7_	Meat	9.523	10.015	10.373	13.665	14.173	15.004	16.811	17.035	17.549
*Y*_2.8_	Fish, shellfish	3.539	3.981	3.484	4.302	4.923	4.957	5.314	5.132	4.723
*Y*_2.9_	Milk and dairy products	6.312	6.122	6.137	8.980	9.346	9.708	9.948	10.505	10.634
*Y*_2.10_	Fats and oils	0.073	0.081	0.085	0.102	0.098	0.114	0.116	0.129	0.131
*Y*_2.11_	Sugars and sweeteners	0.032	0.031	0.026	0.027	0.025	0.024	0.023	0.023	0.023

Source: authors’ development.

**Table A3:** Fat supply quantity (*Y*_3_) per country categories and food groups in 1980–2018, g/capita/day.

Index	Food Group	1980	1985	1990	1995	2000	2005	2010	2015	2018
Low-income economies										
*Y*_3.1_	Cereals	3.354	3.636	3.480	4.103	4.265	4.655	4.894	5.010	4.856
*Y*_3.2_	Fruits	0.514	0.497	0.469	0.456	0.447	0.507	0.585	0.481	0.454
*Y*_3.3_	Vegetables	0.227	0.217	0.219	0.246	0.225	0.246	0.237	0.284	0.294
*Y*_3.4_	Roots, tubers, plantains	0.407	0.367	0.348	0.322	0.386	0.384	0.390	0.443	0.435
*Y*_3.5_	Pulses, seeds, nuts	0.475	0.419	0.422	0.410	0.465	0.497	0.582	0.584	0.560
*Y*_3.6_	Eggs	0.193	0.200	0.216	0.214	0.200	0.235	0.259	0.287	0.274
*Y*_3.7_	Meat	4.535	4.400	4.550	4.871	5.145	5.372	5.634	5.635	5.561
*Y*_3.8_	Fish, shellfish	0.436	0.376	0.385	0.380	0.393	0.445	0.502	0.504	0.525
*Y*_3.9_	Milk and dairy products	2.556	2.376	2.569	2.929	3.066	3.580	3.764	3.318	3.350
*Y*_3.10_	Fats and oils	17.520	18.200	18.985	21.702	21.152	23.339	23.958	23.946	23.965
*Y*_3.11_	Sugars and sweeteners	0.009	0.005	0.003	0.003	0.001	0.001	0.001	0.001	0.001
Lower-middle-income economies										
*Y*_3.1_	Cereals	5.469	5.581	5.562	6.021	6.011	6.199	6.389	6.626	6.749
*Y*_3.2_	Fruits	0.342	0.343	0.328	0.384	0.405	0.408	0.456	0.506	0.554
*Y*_3.3_	Vegetables	0.199	0.210	0.221	0.273	0.333	0.372	0.422	0.491	0.481
*Y*_3.4_	Roots, tubers, plantains	0.259	0.249	0.264	0.291	0.305	0.318	0.338	0.342	0.348
*Y*_3.5_	Pulses, seeds, nuts	0.276	0.298	0.301	0.290	0.322	0.361	0.399	0.382	0.403
*Y*_3.6_	Eggs	0.480	0.488	0.500	0.664	0.703	0.801	0.924	1.041	1.056
*Y*_3.7_	Meat	5.413	5.534	5.956	7.595	8.002	8.665	9.883	10.639	10.763
*Y*_3.8_	Fish, shellfish	0.528	0.604	0.600	0.591	0.738	0.822	0.925	1.020	0.992
*Y*_3.9_	Milk and dairy products	2.999	3.028	3.034	4.752	5.195	5.618	5.791	6.169	6.189
*Y*_3.10_	Fats and oils	17.105	18.531	19.784	23.581	23.553	25.045	26.824	27.195	28.462
*Y*_3.11_	Sugars and sweeteners	0.003	0.004	0.005	0.004	0.004	0.004	0.005	0.005	0.005
Upper-middle-income economies										
*Y*_3.1_	Cereals	3.839	3.879	3.907	4.817	4.947	5.151	5.243	5.512	5.570
*Y*_3.2_	Fruits	0.617	0.607	0.549	0.630	0.694	0.729	0.801	0.937	0.977
*Y*_3.3_	Vegetables	0.245	0.270	0.282	0.412	0.467	0.538	0.564	0.590	0.583
*Y*_3.4_	Roots, tubers, plantains	0.248	0.247	0.230	0.280	0.273	0.288	0.280	0.301	0.290
*Y*_3.5_	Pulses, seeds, nuts	0.308	0.302	0.301	0.308	0.284	0.297	0.279	0.282	0.283
*Y*_3.6_	Eggs	1.124	1.171	1.284	1.673	1.752	2.005	2.200	2.403	2.509
*Y*_3.7_	Meat	9.763	10.367	10.659	14.646	15.093	15.746	17.566	17.871	18.289
*Y*_3.8_	Fish, shellfish	0.878	0.990	0.817	1.000	1.147	1.236	1.373	1.317	1.201
*Y*_3.9_	Milk and dairy products	5.265	5.118	5.496	8.551	8.906	9.327	9.495	9.998	10.091
*Y*_3.10_	Fats and oils	23.698	25.857	27.326	31.645	32.692	36.072	37.594	39.299	40.301
*Y*_3.11_	Sugars and sweeteners	0.008	0.008	0.006	0.007	0.005	0.004	0.004	0.004	0.003

Source: authors’ development.

## Appendix B

**Table A4:** Regressors (*X_n_*) in low-income economies, averaged values, 1980–2018.

Index	Variable	1980	1985	1990	1995	2000	2005	2010	2015	2018
*X*_1_	Gross agricultural production value, USD 1 million	807.51	849.99	1032.41	1483.46	1776.76	2253.33	3170.37	3464.77	3839.63
*X*_2_	Gross per capita agricultural production index, points (2014–2016 = 100)	93.28	88.71	83.62	85.94	88.52	92.13	102.26	99.14	100.49
*X*_3_	Agricultural sector’s share of gross domestic product, %	35.46	36.04	34.66	38.48	35.69	34.26	34.06	32.03	31.72
*X*_4_	Nominal gross national income at current prices per capita, USD	370.59	342.01	388.71	343.26	309.46	417.37	650.22	720.86	709.66
*X*_5_	Commodity price index, food products, points (2000 = 100)	183.87	103.35	121.77	138.85	100.00	128.44	231.56	203.50	194.55
*X*_6_	Commodity price index, agricultural raw materials, points (2000 = 100)	131.11	93.97	128.18	150.36	100.00	129.42	225.72	160.57	165.55
*X*_7_	Consumer price index, points (2010 = 100)	10.75	16.43	18.56	32.97	48.27	69.57	100.00	151.59	216.23
*X*_8_	Total exports of food and agricultural products, USD 1 million	57.91	36.36	43.36	113.49	108.82	157.98	294.58	398.84	492.15
*X*_9_	Total imports of food and agricultural products, USD 1 million	57.14	49.68	25.36	135.63	150.15	300.10	606.59	776.90	885.37
*X*_10_	Currency exchange rate, local currency units/USD	68.77	143.92	152.35	330.02	570.10	683.38	843.73	1115.71	1364.16

Source: authors’ development.

**Table A5:** Regressors (*X_n_*) in lower-middle-income economies, averaged values, 1980–2018.

Index	Variable	1980	1985	1990	1995	2000	2005	2010	2015	2018
*X*_1_	Gross agricultural production value, USD 1 million	6379.55	7697.09	9184.69	11329.70	12871.71	14906.79	17883.47	20824.23	23289.33
*X*_2_	Gross per capita agricultural production index, points (2014–2016 = 100)	70.07	68.23	69.11	75.96	81.57	86.57	94.53	101.02	104.42
*X*_3_	Agricultural sector’s share of gross domestic product, %	23.63	22.26	22.60	24.73	22.66	19.58	17.94	17.21	16.32
*X*_4_	Nominal gross national income at current prices per capita, USD	653.71	584.04	639.42	734.27	737.89	1108.54	1799.39	2143.28	2286.77
*X*_5_	Commodity price index, food products, points (2000 = 100)	183.87	103.35	121.77	138.85	100.00	128.44	231.56	203.50	194.55
*X*_6_	Commodity price index, agricultural raw materials, points (2000 = 100)	131.11	93.97	128.18	150.36	100.00	129.42	225.72	160.57	165.55
*X*_7_	Consumer price index, points (2010 = 100)	14.68	18.18	25.61	40.98	54.20	69.30	100.00	135.63	164.39
*X*_8_	Total exports of food and agricultural products, USD 1 million	340.73	284.16	291.78	751.25	732.55	1262.57	2454.22	3421.79	3946.75
*X*_9_	Total imports of food and agricultural products, USD 1 million	265.27	282.76	335.24	670.25	716.06	1244.13	2564.81	3358.89	3855.78
*X*_10_	Currency exchange rate, local currency units/USD	28.31	61.82	250.01	485.26	867.18	1032.32	1065.55	1225.72	1441.59

Source: authors’ development.

**Table A6:** Regressors (*X_n_*) in upper-middle-income economies, averaged values, 1980–2018.

Index	Variable	1980	1985	1990	1995	2000	2005	2010	2015	2018
*X*_1_	Gross agricultural production value, USD 1 million	14058.49	17690.97	20953.41	28448.85	32940.97	38623.30	44410.85	50580.01	53064.79
*X*_2_	Gross per capita agricultural production index, points (2014–2016 = 100)	84.00	83.88	82.65	93.40	91.98	95.25	97.31	99.96	101.07
*X*_3_	Agricultural sector’s share of gross domestic product, %	11.91	10.95	11.02	15.21	11.46	9.63	8.45	8.14	7.73
*X*_4_	Nominal gross national income at current prices per capita, USD	1491.07	1291.79	1673.61	2340.99	2440.59	3653.83	6040.82	6638.53	7091.28
*X*_5_	Commodity price index, food products, points (2000 = 100)	183.87	103.35	121.77	138.85	100.00	128.44	231.56	203.50	194.55
*X*_6_	Commodity price index, agricultural raw materials, points (2000 = 100)	131.11	93.97	128.18	150.36	100.00	129.42	225.72	160.57	165.55
*X*_7_	Consumer price index, points (2010 = 100)	7.41	10.32	15.27	34.01	53.00	72.17	100.00	136.00	163.24
*X*_8_	Total exports of food and agricultural products, USD 1 million	862.48	915.05	1231.47	2286.01	2226.51	3979.44	7686.02	9504.70	10956.62
*X*_9_	Total imports of food and agricultural products, USD 1 million	391.25	253.92	521.82	1551.42	1544.82	2698.25	5783.36	7202.73	8428.83
*X*_10_	Currency exchange rate, local currency units/USD	28.77	58.96	128.06	287.84	643.25	804.01	766.54	1386.69	1726.99

Source: authors’ development.
